# Vertical Stratification Reduces Microbial Network Complexity and Disrupts Nitrogen Balance in Seasonally Frozen Ground at Qinghai Lake in Tibet

**DOI:** 10.3390/microorganisms13020459

**Published:** 2025-02-19

**Authors:** Ni Zhang, Zhiyun Zhou, Yijun Wang, Shijia Zhou, Jing Ma, Jianqing Sun, Kelong Chen

**Affiliations:** 1 Qinghai Province Key Laboratory of Physical Geography and Environmental Process, College of Geographical Science, Qinghai Normal University, Xining 810008, China; zhangni0224@163.com (N.Z.); 17719649826@163.com (Y.W.); ruye1125@163.com (S.Z.); qhmdmajing@163.com (J.M.); 2 Key Laboratory of Tibetan Plateau Land Surface Processes and Ecological Conservation, Ministry of Education, Qinghai Normal University, Xining 810008, China; 13897423633@163.com; 3 National Positioning Observation and Research Station of Qinghai Lake Wetland Ecosystem in Qinghai, National Forestry and Grassland Administration, Haibei 812300, China; 4 Qinghai Lake National Nature Reserve Administration, Xining 810008, China; sunjq@163.com

**Keywords:** carbon and nitrogen cycle, climate change, elevation gradient, LC-MS, Qinghai–Tibet Plateau, seasonal frozen soil

## Abstract

Global climate change has accelerated the reduction of permafrost regions across different altitude gradients, shortening the duration of the freezing period to varying extents. However, the response of the soil microorganisms of frozen soils along altitude gradients remains unclear. In this study, we employed 16S rRNA sequencing and LC-MS metabolomics to investigate the response of soil microbial communities and soil metabolites to vertical stratification in the permafrost soils of the Qinghai Lake region. The results indicated that Proteobacteria, Firmicutes, and Actinobacteria were key soil bacterial phyla in the permafrost soils of Qinghai Lake during the freezing period, with Proteobacteria and Firmicutes showing significant sensitivity to vertical stratification (*p* < 0.05). The majority of the physicochemical factors exhibited a trend of initially increasing and then decreasing with increasing altitude, whereas pH showed the opposite trend. pH and moisture content were identified as the most important environmental factors influencing soil bacterial community structure. Deterministic processes dominated the assembly of bacterial communities of frozen soils in the Qinghai Lake basin. Co-occurrence network analysis showed that increasing altitude gradients led to a higher average degree of the bacterial network, while reducing network complexity and inter-species connectivity. Soil metabolomics analysis revealed that vertical stratification altered the metabolic profiles of 27 metabolites, with the significantly changed metabolites primarily associated with carbohydrate and amino acid metabolism. In conclusion, the characteristics of the Qinghai Lake permafrost were regulated by regional vertical stratification, which further influenced microbial community structure and soil metabolic characteristics, thereby altering carbon and nitrogen stocks. Specifically, higher altitudes were more favorable for the retention of the carbon and nitrogen stocks of frozen soils in the Qinghai Lake basin.

## 1. Introduction

Qinghai Lake, located in the northeastern part of the Qinghai–Tibet Plateau in China, is one of the largest inland saltwater lakes in the world, characterized by unique climatic, ecological, and geographical characteristics. The region is a typical alpine seasonal freeze–thaw zone, deeply influenced by climate change and extreme environmental conditions [1]. In recent years, with the intensification of climate change, the seasonal patterns in freeze–thaw zones have gradually shifted [2], leading to the release of soil carbon into the atmosphere and accelerating global warming [3,4]. Microorganisms, as the most abundant and ancient forms of life on Earth, play a key role in frozen soil ecosystems [5]. They not only drive soil material cycling and nutrient transformation but are also crucial regulators of soil organic matter decomposition, which in turn governs terrestrial carbon balance [6,7,8]. Changes in freeze–thaw cycles are likely to have profound effects on microbial biodiversity, community structure, and metabolic activity [9,10], further influencing regional carbon dynamics as well as the release and accumulation of global organic carbon [4,11].

The soil freezing period is a key constraint on microbial activity, as the low temperatures and moisture limitations in frozen soil inhibit microbial growth and metabolism [10,12]. With the ongoing development of global warming, permafrost regions are continuously shrinking in many parts of the world, particularly in high-latitude and high-altitude areas, resulting in shorter freezing periods [13,14]. This change will accelerate the microbial decomposition of organic carbon in soils, releasing greenhouse gases (such as carbon dioxide and methane) into the atmosphere and reducing carbon stocks in permafrost regions [15,16]. In addition, the climate and soil heterogeneity of mountain ecosystems exhibit considerable variability over short spatial distances [17]. The high environmental heterogeneity along altitude gradients, such as precipitation and temperature, further leads to variations in microbial characteristics (biomass, respiration, and enzyme activity) along the elevation gradient [18,19]. Therefore, changes in microbial communities during the freezing period and the vertical stratification of metabolic characteristics are likely to directly influence the patterns of carbon accumulation and release in the soil. However, there is a lack of in-depth research on the dynamic changes of microbial communities during the freezing period in permafrost regions and their responses to regional carbon cycling. This is particularly true in regions like the Qinghai–Tibet Plateau, which is rich in carbon stocks but highly vulnerable to climate change, making the interaction between permafrost and carbon dynamics an area worthy of further investigation.

Here, we focus on the Qinghai Lake basin and aim to investigate the microbial community structure and soil metabolic characteristics during the freezing period in the seasonally frozen ground of the Qinghai Lake region. The key aims are as follows: (1) analyze the responses of soil bacterial communities and soil metabolites to vertical stratification, (2) analyze the potential impact of regional vertical stratification during the freezing period on microbial communities and soil metabolites, and (3) analyze the key environmental factors affecting the soil bacterial communities and metabolic potentials.

## 2. Materials and Methods

### 2.1. Overview of the Study Area

The Qinghai Lake basin (36°15′–38°20′ N, 97°50′–101°20′ E) is located in the northeastern part of the Qinghai–Tibet Plateau, characterized by a plateau continental climate and significant vulnerability to climate warming. The basin is encircled by mountains, with a terrain gradient sloping from the southeast to the northwest. Elevations range from 3194 m to 5174 m. A distinctive climatic feature is the large diurnal temperature range, with the annual average temperature varying between −1.5 °C and 1.5 °C. Precipitation is concentrated from May to September, with a peak occurring in July and August. The annual precipitation ranges from 252 mm to 514 mm. In contrast, annual evapotranspiration ranges from 1300 mm to 2000 mm. Vegetation and soil types exhibit significant changes in altitude, ranging from temperate grasslands to alpine meadows, alpine shrublands, and alpine steppes. The main soil types include chestnut soil, alpine meadow soil, and alpine desert soil.

### 2.2. Sample Collection

**Figure 1:**
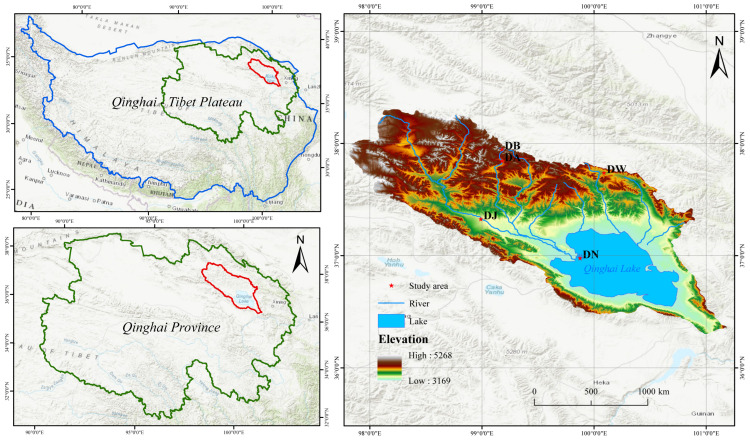
Sampling point distribution.

Based on altitude (3190–4130 m), five representative sampling sites in the Qinghai Lake basin were selected at the end of December 2021, which was in the soil freezing period (Figure 1). The elevation intervals between the sampling sites were approximately 200 m, and they were named in order of elevation as DN, DJ, DW, DA, and DB. At each sampling site, three 1 m × 1 m plots with flat terrains and evenly distributed vegetation were randomly selected. Soil samples from the 0 to 10 cm surface layer were collected using a soil auger (diameter 4.5 cm) with a five-point sampling method. The samples from each plot were mixed thoroughly, passed through a 2 mm sieve to remove visible stones and plant debris, stored in ice bags, and immediately transported to the laboratory. A portion of the soil samples was stored in 10 mL EP tubes at −80 °C for soil DNA extraction. The remaining samples were stored at 4 °C for the analysis of soil physicochemical properties.

### 2.3. Determination of Soil Biogeochemical Properties

The determination of total potassium and available potassium was performed using the NaOH fusion flame photometric method (FP6410 Flame Photometer, Shanghai Instrument Electronics Analytical Co., Ltd., Shanghai, China). Nitrate nitrogen and ammonium nitrogen were measured using the potassium chloride extraction method (Continuous Flow Analyzer, FUTURA, Saint-Raphaël, France). Available phosphorus was determined by the double acid extraction-molybdenum-antimony colorimetric method (UV-1900i UV-Vis spectrophotometer, Shimadzu, Kyoto, Japan), while total phosphorus was measured using the sodium hydroxide fusion-molybdenum-antimony resistance colorimetric method (UV-1900i UV-Vis spectrophotometer, Shimadzu, Kyoto, Japan). Organic matter was determined using the potassium dichromate-concentrated sulfuric acid heating method (Titrette Titrator-4760151, Brand, Wertheim, Germany). Total carbon and total nitrogen were measured using an elemental analyzer (Vario EL III, Elementar Analysis System GmbH, Langenselbold, Germany). Temperature and humidity were measured using a TSS-1K soil temperature and humidity meter (Top Instrument Co., Ltd., Hangzhou, China). Soil pH was measured using a pH meter (Mettler Toledo, Greifensee, Switzerland) after mixing the soil and water in a 1:2.5 ratio.

### 2.4. DNA Extraction and Polymerase Chain Reaction (PCR) Amplification

DNA was extracted from 0.25 g of homogenized soil from each sample using the PowerSoil DNA Isolation Kit (MoBio Laboratories, Carlsbad, CA, USA). The final DNA concentration was measured using a NanoDrop 2000 UV-Vis spectrophotometer (Thermo Scientific, Wilmington, DE, USA). Prokaryotic DNA was amplified using primers 341F (5’-CCTAYGGGRBGCASCAG-3’) and 806R (5’-GGACTACNNGGGTATCTAAT-3’). To minimize PCR bias, three separate reactions were prepared for each sample. The prokaryotic PCR reaction system was conducted according to the protocol described in the literature [20]. The extracted DNA was purified using the PowerClean DNA Kit (MOBIO Laboratories, Carlsbad, CA, USA) and then subjected to paired-end sequencing on the Illumina MiSeq platform (San Diego, CA, USA). The raw data were analyzed using QIIME software (v 1.9.0), with the denoising algorithm DADA2 used to denoise, merge, and de-chimerize the raw sequences.

### 2.5. Metabolite Extraction and UPLC-MS/MS Analysis

The extracts from each sample were mixed in equal volumes to prepare a quality control (QC) sample. The metabolites were extracted simultaneously with the experimental samples to monitor the stability of the analysis. LC-MS analysis was performed using a UHPLC-Q-Orbitrap HRMS^®^ system (Thermo Fisher Scientific™, Wilmington, DE, USA) coupled with an ACQUITY UPLC BEH^®^C18 column (2.1 × 100 mm, 1.7 μm, Waters™, Milford, MA, USA) to analyze metabolites in the soil. In positive ion mode, the mobile phase consisted of 0.1% acetonitrile (B) and 0.1% formic acid in water (A). In negative ion mode, the mobile phase consisted of acetonitrile (B) and 3 mmol/L ammonium formate in water (A). The gradient elution program followed the protocol described in the literature [21]. The eluate from the chromatographic column was introduced into the ionization source of the DuoSpray™ ESI interface and injected into the hybrid QqTOF mass spectrometer, Sciex TripleTOF 6600 LC-MS system (AB Sciex, Darmstadt, Germany). The analysis was performed in SWATH (Sequential Window Acquisition of All Theoretical Mass Spectra) mode with positive polarity, and data were collected in sequential windows. The system was controlled using Analyst™ TF software version 1.8 [21].

### 2.6. Statistical Analysis

To investigate the correlations and differential characteristics between the data, the alpha diversity indices (Chao1, ACE, Simpson, and Shannon indices) and Beta diversity indices (Non-metric Multidimensional Scaling (NMDS), Partial Least Squares Discriminant Analysis (PLS-DA), and Analysis of Similarities (ANOSIM)) were calculated using the MicrobiotaProcess package (v1.18.0). The linkET package (v0.0.7.4) was used to calculate data correlations and generate a correlation network heatmap. The betaNTI index was calculated using the Picante package (v1.8.2) based on a null model, and the Raup–Crick (RCbray) index was calculated using the microeco package (v1.11.0) to determine the relative importance of deterministic and stochastic processes in community assembly. The psych package (v2.4.12) was used to calculate the correlations between microbes within the same group, and the aov function of stats package (v4.4.1) was used for analysis of variance (ANOVA) to assess the significance of microbial community composition, community diversity, and soil physicochemical properties across different groups. All analyses were performed in R software (v4.4.1). The network diagrams were generated using Gephi 0.9.7 and Cytoscape 3.9.1.

## 3. Results

### 3.1. Environmental Factors

**Figure 2:**
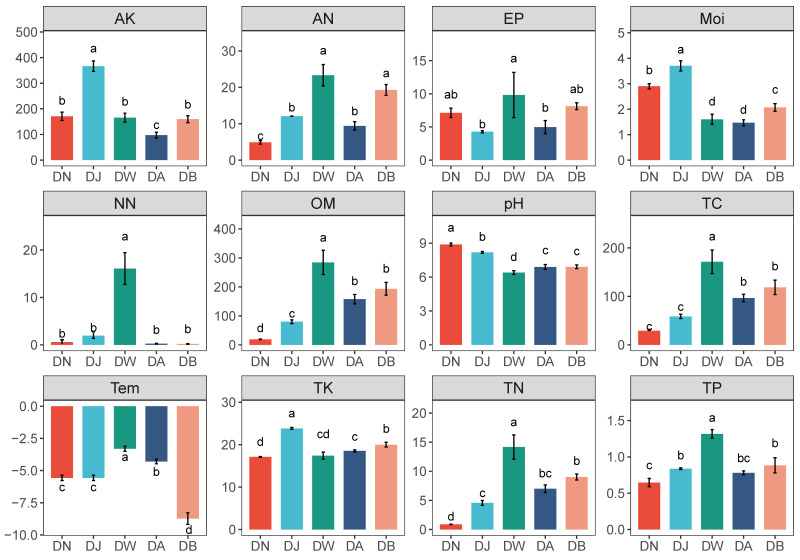
Physicochemical properties of frozen soil samples under vertical stratification in the Qinghai Lake basin. Letters a, b, c, and d indicate significance levels; the same letter denotes no significant difference (*p* > 0.05), while different letters indicate significant differences (*p* < 0.05). Tem: soil temperature, Moi: soil moisture, TN: total nitrogen, TC: total carbon, pH: soil pH, TP: total phosphorus, TK: total potassium, AN: ammonium nitrogen, NN: nitrate nitrogen, EP: effective phosphorus, AK: available potassium, OM: organic matter.

Regional vertical stratification caused significant differences in soil environmental factors in the Qinghai Lake basin (Figure 2, *p* < 0.05). Soil pH showed a trend of initially decreasing and then increasing with elevation, reaching its lowest value at DW (Figure 2). Most physicochemical factors showed a trend of initially decreasing and subsequently increasing with increasing altitude. Among them, AK, TK, and Moi were highest during DJ, while AN, NN, TP, Tem, and EP were highest during DW (Figure 2). Additionally, the contents of TC, TN, and OM showed a gradual increase with the rising altitude gradient (Figure 2).

### 3.2. Bacterial Diversity

**Figure 3:**
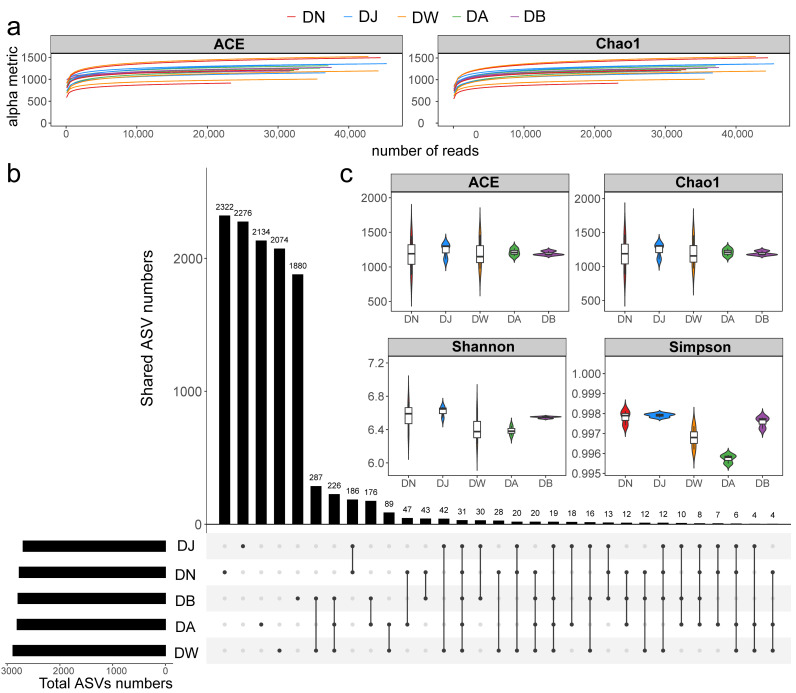
Soil bacterial sequencing characteristics under vertical stratification of frozen soils in the Qinghai Lake basin. (**a**) Sample rarefaction curves; (**b**) ASV distribution across groups; and (**c**) alpha diversity indices.

The sequencing data from the samples were considered reasonable (Figure 3a), with the number of bacterial community sequences obtained from frozen soils in the Qinghai Lake basin ranging from 32,567 to 59,918. Only 31 ASVs (Amplicon Sequence Variants) were shared across all groups (Figure 3b). The total number of ASVs for DN, DJ, DW, DA, and DB was 2771, 2698, 2890, 2811, and 2795, respectively, with the number of unique ASVs being 2322, 2276, 2074, 2134, and 1880, respectively (Figure 3b). Soil heterogeneity in the Qinghai Lake basin was smaller than the elevation differences, and the microbial communities in frozen soils were influenced by the elevation gradient (Figure S1a). NMDS, Adonis, and ANOSIM further confirmed the validity of the groupings, showing that vertical stratification caused significant differences in the bacterial communities (*p* < 0.001; Figure S1b). However, the response of bacterial community alpha diversity to the elevation gradient was not significant (Figure 3c, *p* > 0.05), although it generally exhibited a trend of increasing and then decreasing with increasing elevation.

### 3.3. Bacterial Community Structure

**Figure 4:**
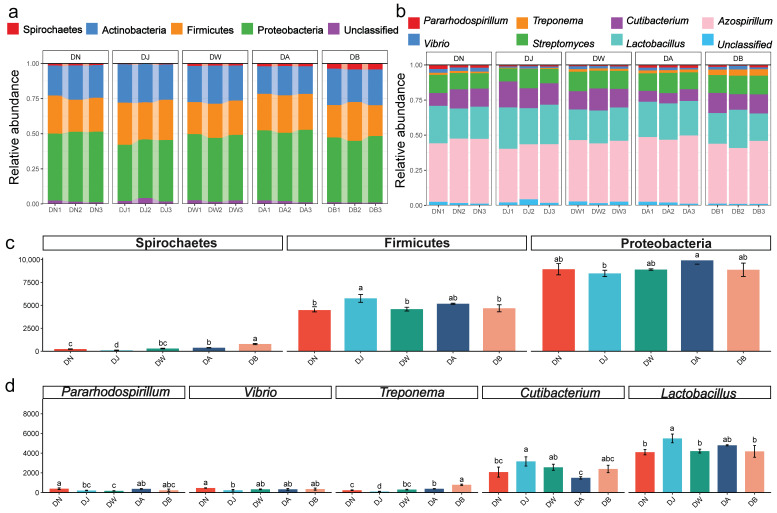
Bacterial community composition under vertical stratification of frozen soils in the Qinghai Lake basin. (**a**) Dominant bacterial phyla at the phylum level; (**b**) dominant bacterial genera at the genus level; (**c**) differential bacterial phyla at the phylum level; and (**d**) differential bacterial genera at the genus level. Letters a, b, c, and d indicate statistical significance. Identical letters indicate no significant difference (*p* > 0.05), while different letters indicate significant differences (*p* < 0.05).

The dominant bacterial phyla (Figure 4a) in the Qinghai Lake basin soil were Proteobacteria (46.57%), Firmicutes (25.43%), Actinobacteria (24.02%), and Spirochaetes (1.87%), together accounting for more than 97% of the total sequences. The relative abundances of Proteobacteria, Firmicutes, and Spirochaetes were significantly influenced by the elevation gradient (*p* < 0.05) (Figure 4c). The first two phyla exhibited a trend of increasing and then decreasing with elevation, with the highest relative abundance of Proteobacteria observed at DA, and the highest relative abundance of Firmicutes found at DJ. The relative abundance of Spirochaetes gradually increased with elevation. At the genus level, the dominant microbial groups (Figure 4b) were *Azospirillum* (40.92%), *Lactobacillus* (23.22%), *Cutibacterium* (11.87%), *Streptomyces* (11.69%), *Treponema* (1.85%), *Vibrio* (1.75%), and *Pararhodospirillum* (1.43%), which together accounted for over 92% of the total sequences. Five of these genera showed statistically significant differences (*p* < 0.05) (Figure 4d). The relative abundances of *Lactobacillus* and *Cutibacterium* followed a trend of first increasing and then decreasing with elevation, peaking at DJ. In contrast, the relative abundances of *Treponema*, *Vibrio*, and *Pararhodospirillum* showed the opposite trend, with Treponema and Vibrio reaching their lowest values at DJ, and *Pararhodospirillum* being lowest at DW.

### 3.4. Bacterial Community Function

**Figure 5:**
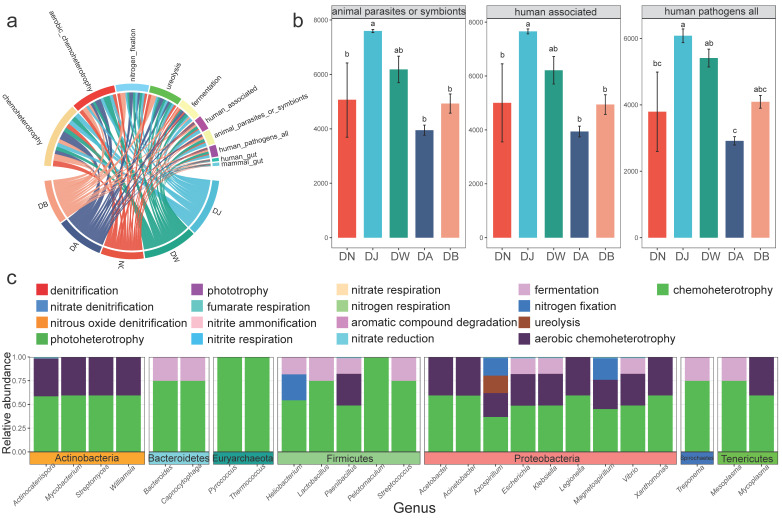
Bacterial functional groups under vertical stratification of frozen soils in the Qinghai Lake basin. (**a**) Major functional groups (Top 10); (**b**) functional groups with inter-group differences; and (**c**) carbon and nitrogen cycling-related functional groups and microbial communities. Letters a, b, and c indicate statistical significance. Identical letters indicate no significant difference (*p* > 0.05), while different letters indicate significant differences (*p* < 0.05).

The results of bacterial functional classification annotation (Figure 5a) showed that the microbial community ecological functions of frozen soils in the Qinghai Lake basin were classified into 15 functional groups (relative abundance > 0.1%). The functional roles of the bacterial community were primarily centered on chemoheterotrophy (26.28%), aerobic chemoheterotrophy (17.95%), nitrogen fixation (13.24%), ureolysis (13.22%), fermentation (8.86%), human associated (5.74%), animal parasites or symbionts (5.74%), human pathogens all (4.60%), human gut (1.27%), and mammal gut (1.27%), collectively accounting for more than 98% of the total relative abundance. Statistical analysis indicated that vertical stratification had little effect on the functional groups involved in carbon and nitrogen cycling (*p* > 0.05), but significantly altered the relative abundances of the functional group human associated, animal parasites or symbionts, and human pathogens all (Figure 5b, *p* < 0.05). These groups exhibited a consistent trend of first increasing and then decreasing with elevation, reaching their highest values at DJ. The reverse annotation of bacterial communities related to carbon and nitrogen cycling revealed that twenty-five genus-level bacterial groups belonging to seven phyla were primarily involved in the carbon and nitrogen cycling processes (Figure 5c). All of these groups were associated with the chemoheterotrophy process, and most were closely linked to the aerobic chemoheterotrophy and fermentation processes.

### 3.5. Correlation Analysis

**Figure 6:**
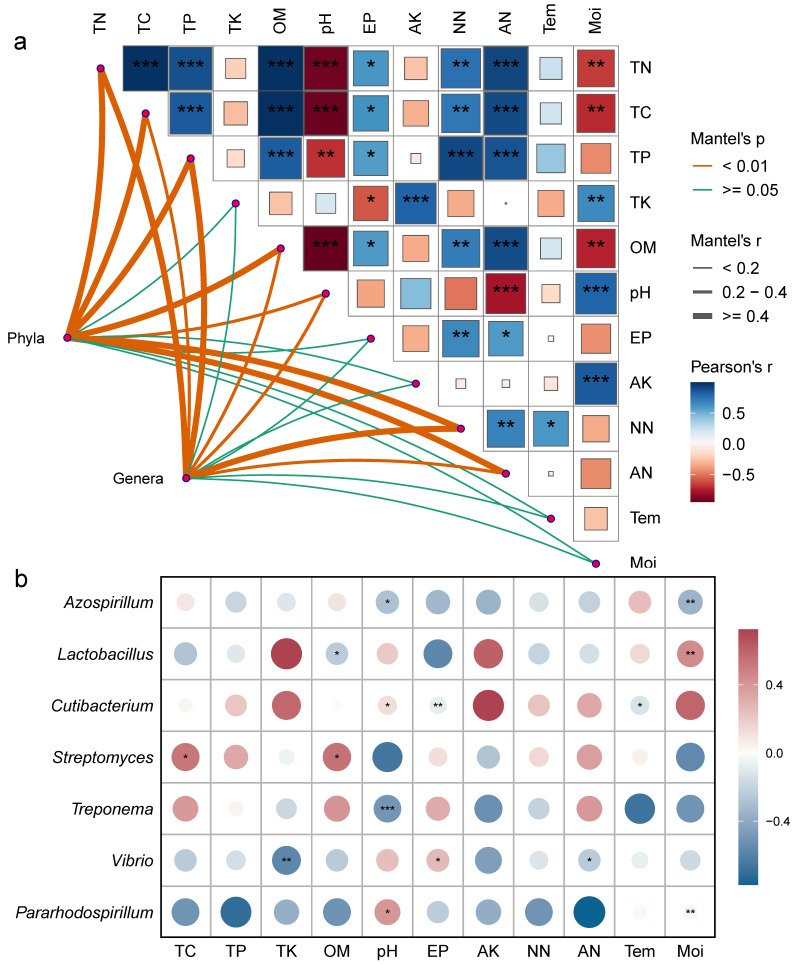
Correlation analysis of frozen soil samples under vertical stratification in the Qinghai Lake basin. (**a**) Correlation network between bacterial community structure and physicochemical factors. (**b**) Correlation heatmap between dominant bacterial genera and physicochemical factors. * Indicates *p* < 0.05, ** indicates *p* < 0.01, *** indicates *p* < 0.001. Tem: soil temperature, Moi: soil moisture, TN: total nitrogen, TC: total carbon, pH: soil pH, TP: total phosphorus, TK: total potassium, AN: ammonium nitrogen, NN: nitrate nitrogen, EP: effective phosphorus, AK: available potassium, OM: organic matter.

Mantel correlation analysis revealed (Figure 6a) that both bacterial phyla and genera were significantly correlated with soil carbon, nitrogen, organic matter, total phosphorus content, and pH. Environmental factors mostly exhibited significant positive correlations, while pH showed a negative correlation with carbon, nitrogen, phosphorus, and organic matter content (Figure 6a). To further clarify the relationship between environmental factors and bacterial communities, a correlation heatmap was generated (Figure 6b, *p* < 0.05). The results showed significant positive correlations between *Streptomyces* and TC and OM; pH and *Pararhodospirillum* and *Cutibacterium*; EP and *Vibrio*; and *Lactobacillus* and Moi. Significant negative correlations were observed between *Azospirillum* and pH and Moi; *Lactobacillus* and OM; *Cutibacterium* and EP and Tem; *Treponema* and pH; and *Vibrio* and TK and AN.

### 3.6. Bacterial Community Construction

**Figure 7:**
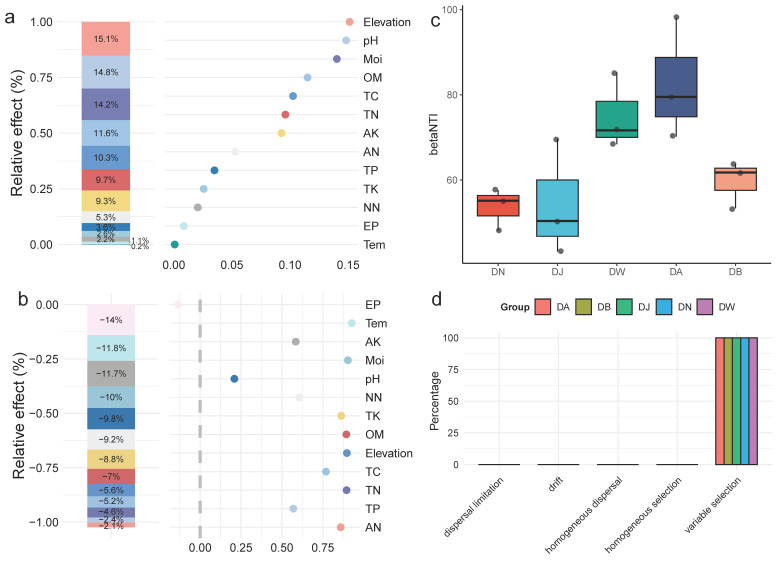
Factors influencing the bacterial community and community assembly processes of frozen soils in the Qinghai Lake basin. (**a**) Hierarchical partitioning analysis of factors affecting bacterial community structure; (**b**) hierarchical partitioning analysis of factors affecting bacterial community diversity; (**c**) distribution of the βNTI index; and (**d**) bacterial community assembly processes. Tem: soil temperature, Moi: soil moisture, TN: total nitrogen, TC: total carbon, pH: soil pH, TP: total phosphorus, TK: total potassium, AN: ammonium nitrogen, NN: nitrate nitrogen, EP: effective phosphorus, AK: available potassium, OM: organic matter.

To analyze the specific impact of environmental factors on the bacterial community structure of frozen soils in the Qinghai Lake basin, a generalized linear model was used for hierarchical partitioning analysis (Figure 7a,b). The results showed that the elevation gradient, pH, moisture (Moi), organic matter (OM), total carbon (TC), total nitrogen (TN), and available potassium (AK) explained 85% of the variation in the bacterial community structure during the freezing period in Qinghai Lake frozen soils. The community structure was most strongly influenced by the elevation gradient, with pH and Moi being the most important environmental factors, followed by OM and TC (Figure 7a). The response of bacterial community diversity to the elevation gradient was not significant, with effective phosphorus (EP) being the primary driver of community diversity. Temperature (Tem), available potassium (AK), and moisture (Moi) were also important driving factors (Figure 7b). The calculation of βNTI values for the bacterial communities under vertical stratification in the Qinghai Lake basin indicated that, at this regional scale, deterministic processes dominate the clustering of the frozen soil bacterial communities in the Qinghai Lake basin (|βNTI| > 2) (Figure 7c). The further calculation of RCbray distinguished the relative impacts of dispersal limitation, drift, homogenizing dispersal, and selection on community dynamics (Figure 7d). The results indicated that vertical stratification in the Qinghai Lake basin did not alter the key processes involved in the construction of frozen soil bacterial communities. Heterogeneous selection dominated the formation of frozen soil bacterial communities, with its contribution remaining at 100% across different elevation gradient treatments.

### 3.7. Soil Metabolites

**Figure 8:**
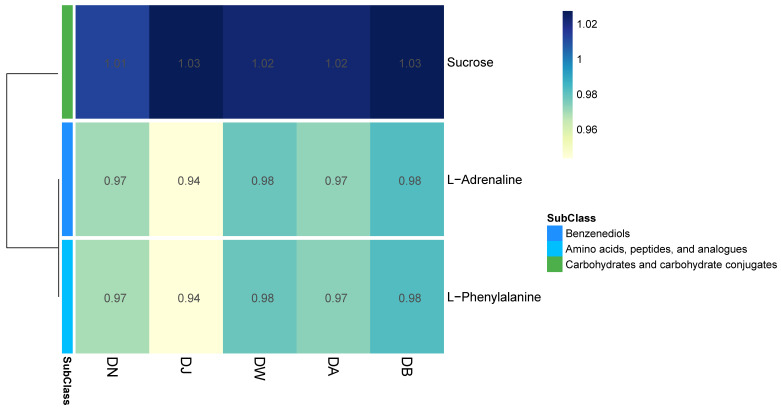
Heatmap of differential metabolites under vertical stratification of frozen soils in the Qinghai Lake basin.

In the principal component analysis (PCA) of the LC-MS untargeted metabolomics, the QC samples, after being mixed, clustered tightly (Figure S1c), indicating the reproducibility of the experimental method and the stability of the instrumental analysis system. Partial least squares discriminant analysis (PLS-DA) revealed the differences in the metabolic profiles among the groups (Figure S1d), showing statistically significant differences between groups, with relatively small differences observed between the DW, DA, and DB groups. Vertical stratification significantly influenced the relative abundances of three major metabolites, which belong to three subclasses, with relative abundances greater than 0.01 (Figure 8). The relative abundance of sucrose (carbohydrates and carbohydrate conjugates) was highest, showing a trend of initially increasing and then decreasing with elevation. In contrast, the relative abundances of L-Adrenaline (benzenediols) and L-Phenylalanine (amino acids, peptides, and analogues) followed a “hump-shaped” trend with increasing elevation (Figure 8).

### 3.8. Correlation Analysis Between Soil Metabolites and Dominant Bacterial Communities

**Figure 9:**
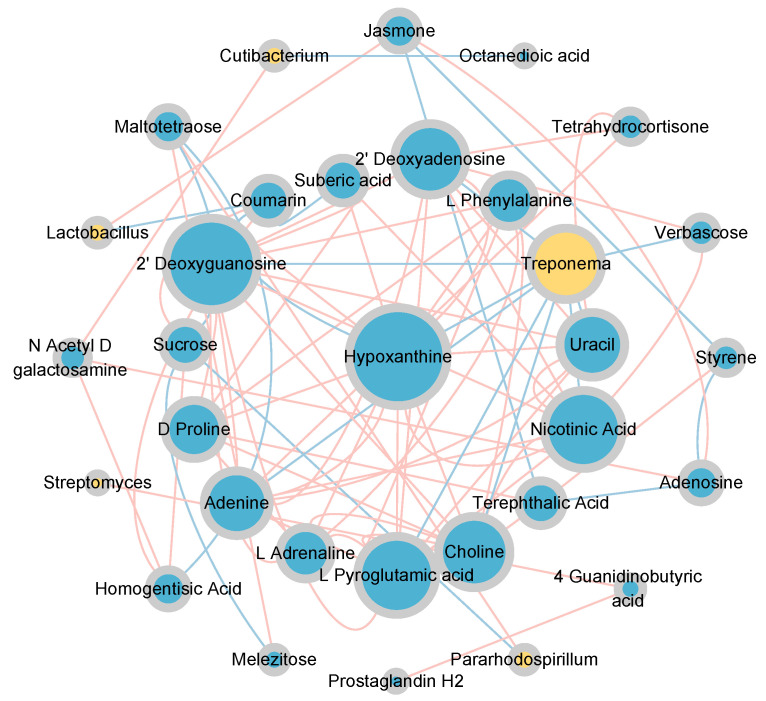
Correlation network between bacterial communities and differential metabolites under vertical stratification of frozen soils in the Qinghai Lake basin. Node size represents degree, and node color indicates category, yellow for genus-level bacterial communities and blue for metabolites; edge color indicates correlation type, red for positive correlation and blue for negative correlation.

The topological relationships between dominant bacterial genera (relative abundance > 0.01) and differential metabolites (relative abundance > 0.001, *p* < 0.05) were shown in Figure 9. *Lactobacillus* exhibited a significant positive correlation with Jasmone and a significant negative correlation with Coumarin. *Cutibacterium* was significantly positively correlated with N-Acetyl-D-galactosamine and significantly negatively correlated with Octanedioic acid. *Streptomyces* exhibited a significant positive correlation with 4-Guanidinobutyric acid. *Treponema* exhibited significant negative correlations with Verbascose, L-Pyroglutamic acid, Choline, Nicotinic Acid, Uracil, 2’-Deoxyguanosine, Hypoxanthine, Adenine, and 2’ Deoxyadenosine. *Pararhodospirillum* was significantly positively correlated with Maltotetraose and significantly negatively correlated with Sucrose. Among them, *Streptomyces* (Actinobacteria), *Lactobacillus* (Firmicutes), and *Treponema* (Spirochaetes) were closely associated with the carbon and nitrogen cycles (Figure 5c).

### 3.9. Bacterial Interaction Networks

**Figure 10:**
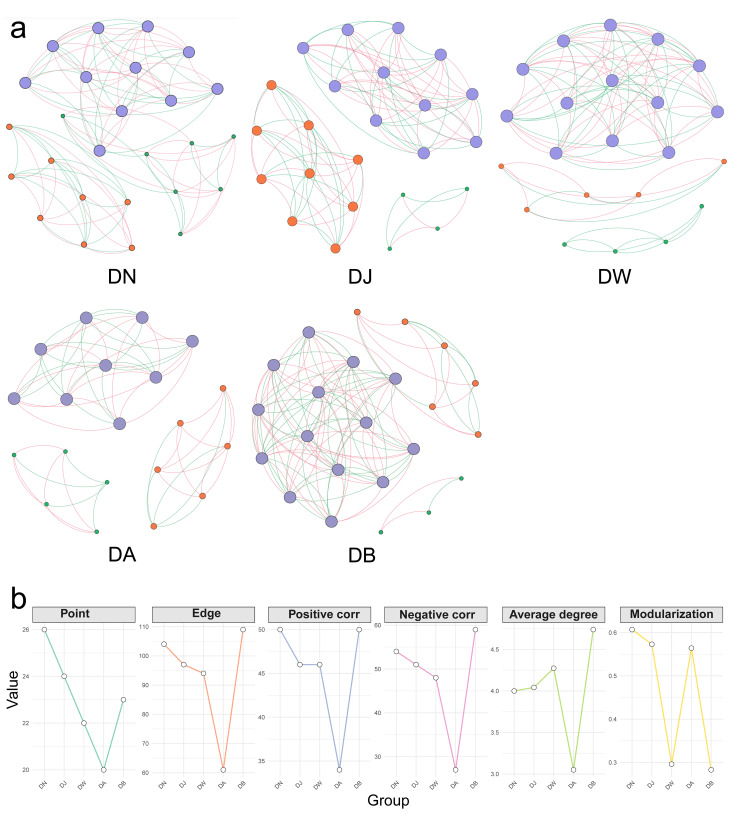
Bacterial network patterns (**a**) and key topological characteristics (**b**) under vertical stratification of frozen soils in the Qinghai Lake basin. The size of the nodes represents the degree; the node color indicates different modules; the edge color represents positive or negative correlations, with red indicating positive correlations and green indicating negative correlations.

Vertical stratification caused differences in the trends of changes in the topological properties of the bacterial interaction network in frozen soils (Figure 10, Table 1). Except for DB, the increase in the elevation gradient consistently led to a decrease in the complexity of the bacterial network in frozen soils, along with a continuous reduction in the connectivity between species (Figure 10, Table 1). This was primarily reflected in the decrease in the total number of nodes and edges in the bacterial network with increasing elevation, as well as a downward trend in both positive and negative correlations (Figure 10, Table 1). In addition, except for DA, the average degree of the bacterial network increased with elevation. However, the trend in modularity did not follow a clear pattern. Modularity was lowest in DW and DB, where the inter-species connections were weaker compared to other elevation gradients (Figure 10, Table 1).

## 4. Discussion

### 4.1. Response of Soil Microbial Community Structure to Altitudinal Gradients During the Freezing Period

The dominant bacterial phylum in the Qinghai Lake basin was Proteobacteria, and vertical differentiation did not alter this dominance. A study by Wang et al. [22] in northwestern Sichuan also indicated that, regardless of altitude, Proteobacteria was the predominant bacterial phylum in the soil. However, the relative abundance of Proteobacteria in frozen soils was significantly influenced by the altitudinal gradient, showing an initial increase followed by a decrease with rising altitude. Recent studies have shown that the abundance of Proteobacteria was higher at lower altitudes compared to higher altitudes [23]. Previous research has also shown that the abundance of Proteobacteria in soil samples decreased with increasing altitude, as reported by Xiang et al. [24]. In contrast, a study by Cong et al. [25] on the Qinghai–Tibet Plateau found that the relative abundance of Proteobacteria significantly decreased with decreasing altitude. This discrepancy may be attributed to soil heterogeneity and biological interactions [26]. In addition, the relative abundance of the dominant genera *Lactobacillus* and *Cutibacterium* at the genus level also exhibited a trend of initially increasing and then decreasing with the rise in altitude. Changes in microbial communities often have an impact on ecosystem processes and functions [27,28]. However, in this study, vertical differentiation did not significantly alter the relative abundance of functional groups involved in the carbon and nitrogen cycles. Soil properties have long been considered key determinants of soil bacterial community structure [29]. This study found that the bacterial community structure during the freezing period in Qinghai Lake frozen soils was most influenced by the altitude gradient, with pH being the most important environmental factor. In contrast, Xiang et al. [24] identified available phosphorus (AP) as the most significant variable influencing bacterial community structure, with altitude as the second most important factor. Similarly, Huang et al. [30] found that soil pH had a significant impact on the relative abundance of bacteria at different altitudes in northwestern Sichuan, which is consistent with the findings of this study. In addition, the bacterial community assembly process of frozen soils in the Qinghai Lake basin was dominated by heterogeneous selection in deterministic processes, driving vertical differentiation. A study by Zhu et al. [31] in the Gongga Mountain forest ecosystem found that the aggregation and symbiotic patterns of soil bacterial communities were determined by altitude rather than season, with soil bacterial community composition being primarily governed by deterministic processes. Similarly, Shen et al. [32] found that the bacterial community assembly process along the altitudinal gradient on the Qinghai–Tibet Plateau was dominated by deterministic processes. Network analysis further revealed the complex relationships between microbial taxa [33,34]. The results showed that the average degree of the bacterial network increased with rising altitude, indicating a higher degree of habitat heterogeneity for microorganisms in high-altitude soils [35]. As the altitude gradient increased, the complexity of the bacterial network during the freezing period and the connectivity between species continuously decreased. This suggests that the connections between soil bacteria are stronger at lower altitudes than at higher altitudes [36,37].

### 4.2. Soil Metabolites at Different Altitudinal Gradients During the Freezing Period and Their Interactions with Microorganisms

Vertical differentiation in seasonally frozen ground at Qinghai Lake during the freezing period led to significant changes in the relative abundance of twenty-seven differential metabolites, three of which were identified as the major differential metabolites (relative abundance > 0.01). These metabolites, as energy sources for bacterial metabolism [38], typically influence the structure and function of microbial communities [39,40]. Sucrose, a carbohydrate subclass, showed a trend of initially increasing and then decreasing with rising altitude. L-Adrenaline (benzenediols) and L-Phenylalanine (amino acids, peptides, and analogues) were closely related to nitrogen cycling processes. Their changes in relative abundance exhibited a similar “hump-shaped” trend with increasing altitude. L-Phenylalanine, as an amino acid, was closely associated with nitrogen transformation and is typically directly absorbed by soil microorganisms and plants [41]. Therefore, the vertical differentiation of seasonally frozen ground at Qinghai Lake may disrupt the nitrogen balance in the soil ecosystem and reduce nitrogen availability [42]. To further elucidate the relationship between soil microorganisms and differential metabolites, we constructed a co-occurrence network. The results showed that 16 metabolites, including Jasmone, Coumarin, N-Acetyl-D-galactosamine, Octanedioic acid, 4-Guanidinobutyric acid, Verbascose, L-Pyroglutamic acid, Choline, Nicotinic Acid, Uracil, 2’-Deoxyguanosine, Hypoxanthine, Adenine, 2’-Deoxyadenosine, Maltotetraose, and Sucrose, were significantly correlated with the dominant bacterial taxa. Maltotetraose and Sucrose were significantly correlated with *Pararhodospirillum*, while the remaining metabolites were mostly significantly negatively correlated with the key microbial communities involved in carbon and nitrogen cycling. The significant positive correlation between *Streptomyces* and 4-Guanidinobutyric acid may be due to extracellular enzymes secreted by the microorganisms [43,44]. In summary, the vertical differentiation of the freezing period in seasonally frozen ground altered soil metabolism and impacted microbial communities, thereby influencing carbon and nitrogen cycling processes [45].

### 4.3. Response of Soil Microbial Community Diversity to Altitudinal Gradients During the Freezing Period

In the alpine ecosystem during the freezing period, within an altitude range of 900 m (3190–4130 m), bacterial community alpha diversity initially increased and then decreased with rising altitude, although the changes were not significant. A study by Wang et al. [22] on the altitudinal patterns of soil bacterial communities on Sichuan mountain slopes showed that there were no significant differences in soil bacterial alpha diversity across different altitudes, which supports the findings of the present study. However, this result differs from the distribution patterns observed in previous studies. For example, Shen et al. [46] reported a U-shaped distribution of soil bacterial diversity along the altitudinal gradient on Mount Kilimanjaro. Similarly, Nottingham et al. [47] explored the species diversity of bacteria and fungi along an altitudinal gradient in the Andes (up to 3.5 km), finding that bacterial diversity decreased with increasing altitude. Bahram et al. [48] investigated the structure and function of global surface soil microbial communities, and their results indicated that bacterial diversity exhibited a hump-shaped pattern with increasing altitude. In contrast, Huang et al. [30] conducted a study in the Xuebaoding Nature Reserve and found that the bacterial community richness index increased with altitude, while the Shannon index decreased with increasing elevation. This study also identified EP, Tem, AK, and Moi as important drivers of bacterial community diversity. However, a study by Zeng et al. [49] on freshwater lake sediments found that pH was the primary factor influencing the decline in bacterial diversity along the altitudinal gradient in freshwater lake sediments. Similarly, Shen et al. [32] conducted research in high-altitude regions of the Qinghai–Tibet Plateau and showed that pH, MAT, and the C:N ratio were significant factors affecting bacterial diversity (*p* < 0.05). Song et al. [50] studied the bacterial community in the subtropical forests of Mount Huang and found that bacterial diversity was primarily associated with surface soil pH. Cong et al. [25] examined the altitudinal patterns of soil bacteria in alpine meadows on the Qinghai–Tibet Plateau and concluded that soil bacterial diversity was closely related to soil moisture, pH, and SOC. This discrepancy may be attributed to the amplified heterogeneity of surface soil environments and the strong selective forces of biological interactions at different altitudinal scales and across distinct ecosystems [26,51,52].

## 5. Conclusions

In this study, we investigated the effects of regional vertical differentiation on the soil microbial community, soil physicochemical properties, and soil metabolites during the freezing period in the Qinghai Lake basin. Altitude gradient was the primary factor influencing the bacterial community structure in seasonally frozen ground at Qinghai Lake, with Proteobacteria being the dominant group significantly regulated by altitude. pH was the most important factor controlling the bacterial community structure. In the Qinghai Lake basin, microbial habitat heterogeneity in frozen soils was higher in high-altitude areas, whereas bacterial connections were stronger in low-altitude soils. Vertical differentiation significantly altered the relative abundance of Sucrose and L-Phenylalanine, suggesting that changes in altitude may affect the carbon storage in Qinghai Lake frozen soils and disrupt the existing nitrogen balance. The results of this study are of significant importance for understanding the response of soil microbial community structure to vertical variation in alpine regions. These data provide deeper insights into the driving mechanisms of global climate change on soil microbial communities and alpine ecosystems.

## Figures and Tables

**Table 1:** Topological parameters of the bacterial network in frozen soils under vertical stratification in the Qinghai Lake basin.

Group	Average Degree	Modularization	Edge	Negative Correlation	Positive Correlation	Point
DN	4.000	0.607	104	54	50	26
DJ	4.042	0.573	97	51	46	24
DW	4.273	0.296	94	48	46	22
DA	3.050	0.564	61	27	34	20
DB	4.739	0.283	109	59	50	23

## Data Availability

The data that support the findings of this study are openly available in NCBI at https://www.ncbi.nlm.nih.gov/, reference number PRJNA1188374.

## Supplementary Materials

The following supporting information can be downloaded at: https://www.mdpi.com/article/10.3390/microorganisms13020459/s1, Figure S1: β-diversity analysis. (a) principal component analysis of microorganisms; (b) grouping validity verification; (c) principal component analysis of metabolites; and (d) PLS-DA partial least square analysis of metabolites. *** indicates *p* < 0.001.
