# Supplementary figures and images for: Vertical Stratification Reduces Microbial Network Complexity and Disrupts Nitrogen Balance in Seasonally Frozen Ground at Qinghai Lake in Tibet
Source: Microorganisms. 2025 Feb 19;13(2):459. doi: 10.3390/microorganisms13020459 (PMC11858239; doi:10.3390/microorganisms13020459)

**a**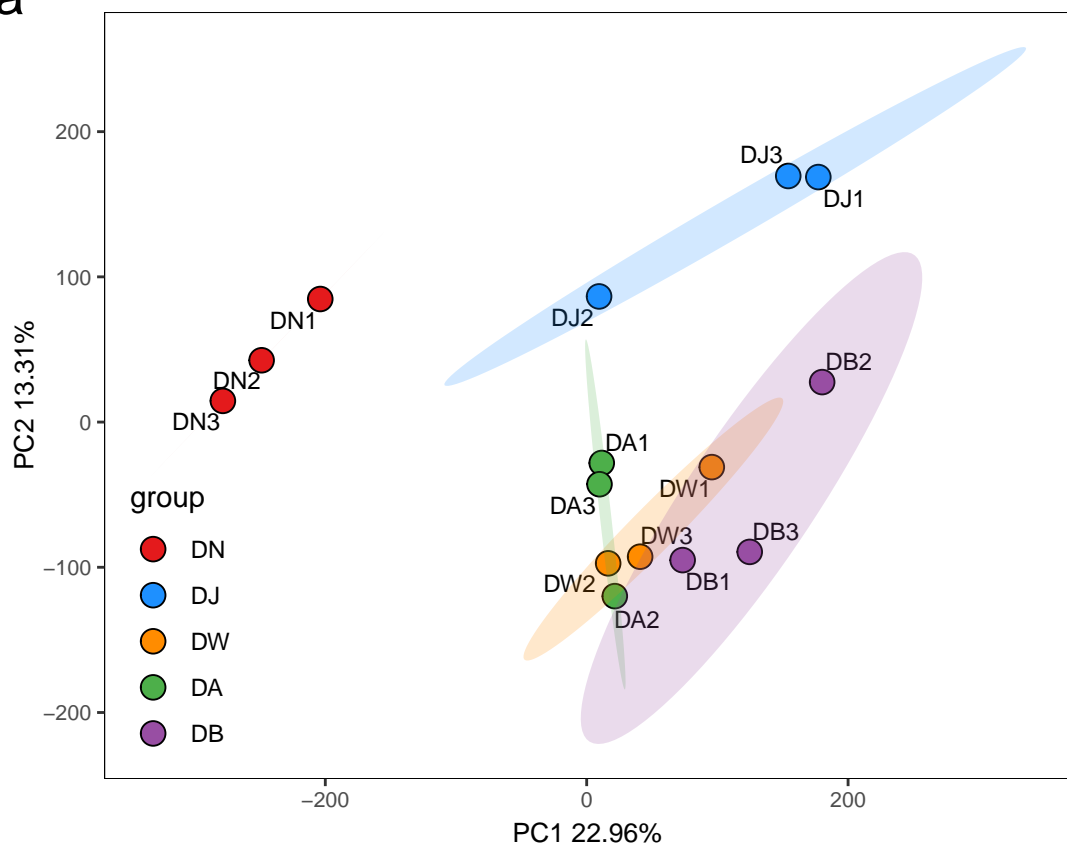**b**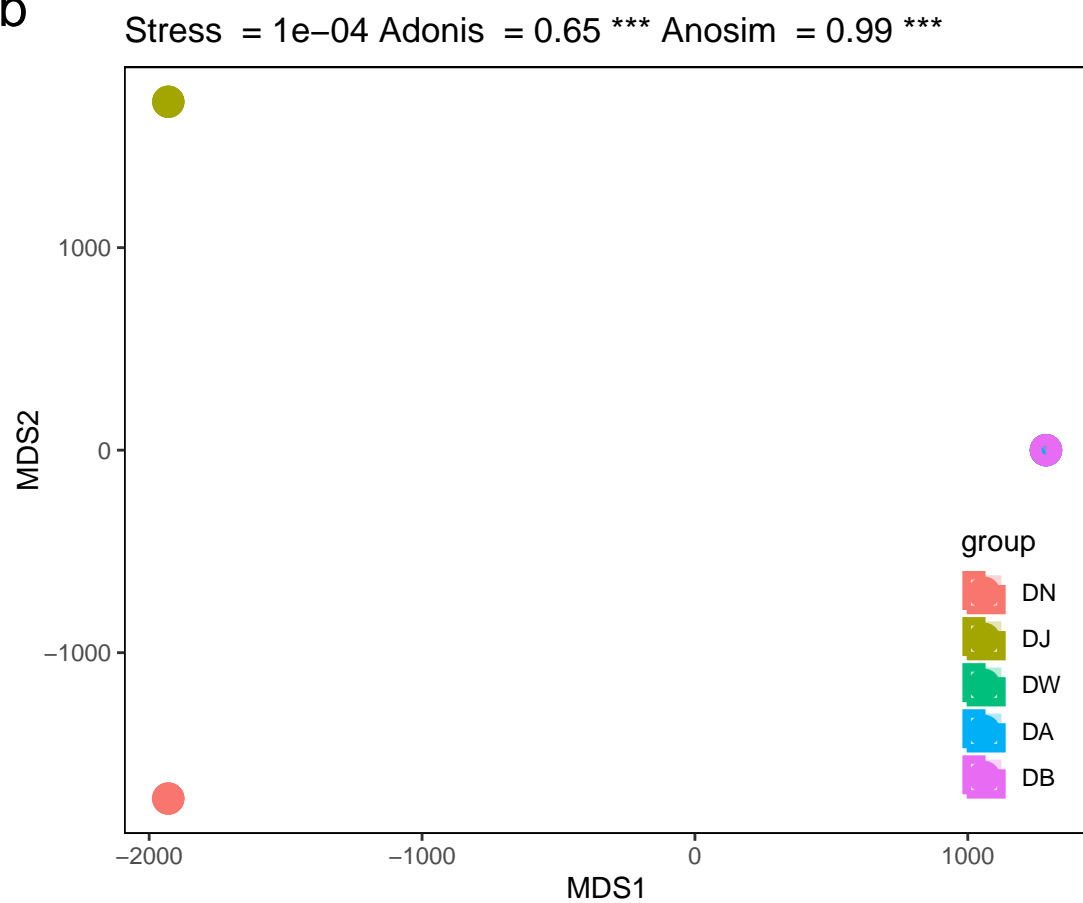**c**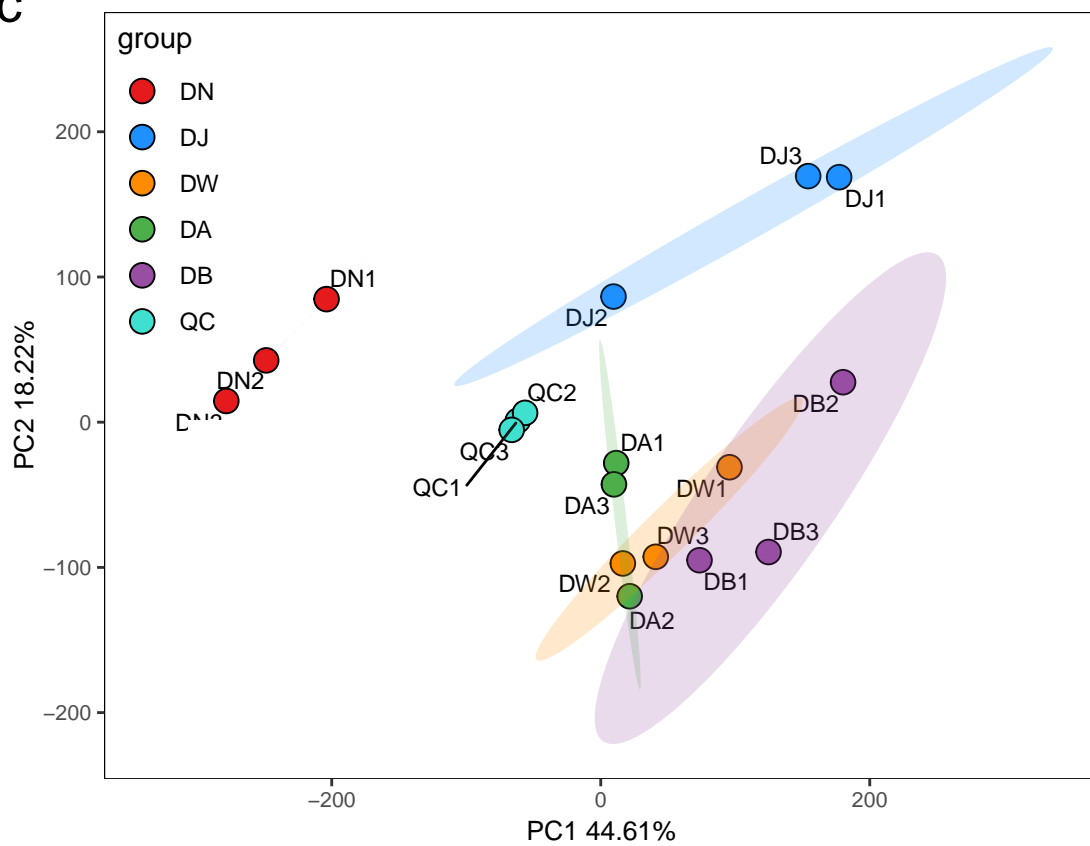**d**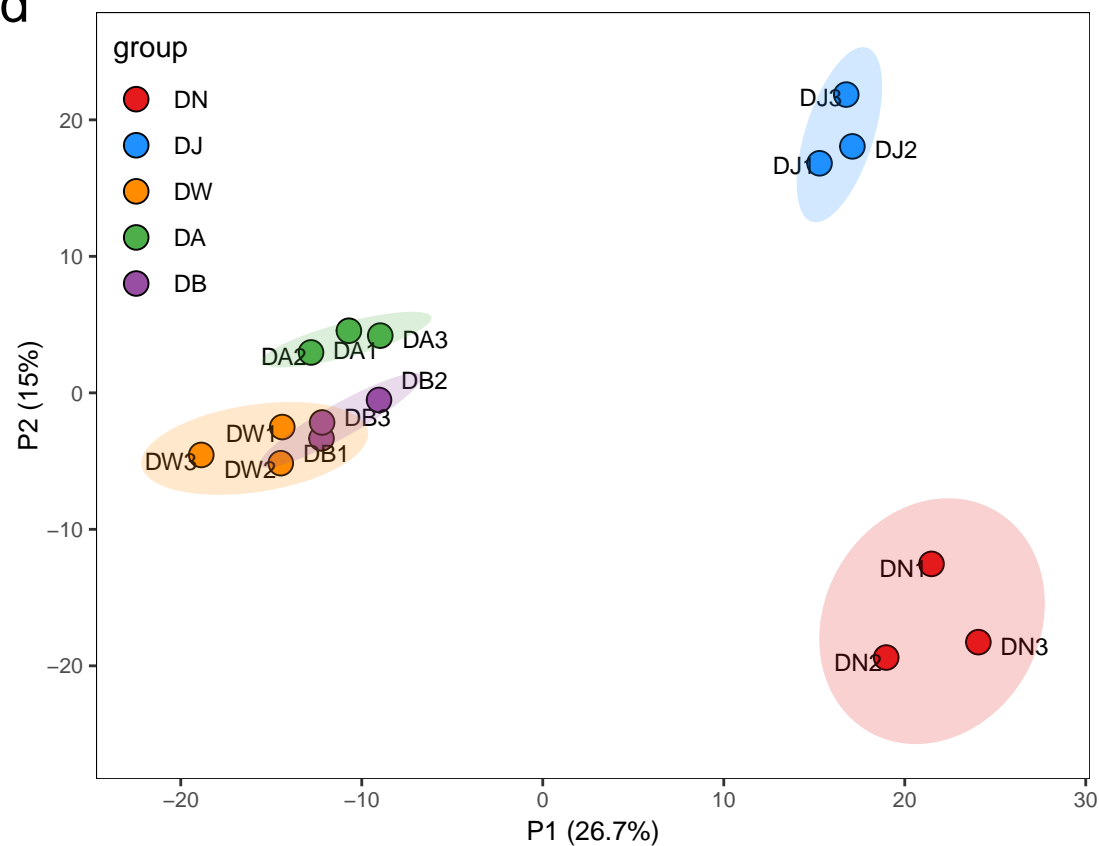

Supplement: Supplementary file 1 [file microorganisms-13-00459-s001.zip › microorganisms-3441871-supplementary.pdf]
